# Clinical characteristics depending on magnetic resonance imaging patterns in idiopathic isolated optic neuritis

**DOI:** 10.1038/s41598-023-28904-6

**Published:** 2023-02-04

**Authors:** Sang Min Park, Ungsoo Samuel Kim

**Affiliations:** 1 Kim’s Eye Hospital, Seoul, South Korea; 2 Department of Ophthalmology, Chung-Ang University College of Medicine, Seoul, South Korea; 3 Chung-Ang University Gwangmyeong Hospital, Gwangmyeong-si, Gyeonggi-do South Korea

**Keywords:** Diseases, Signs and symptoms

## Abstract

To investigate differences in clinical features based on magnetic resonance imaging (MRI) in idiopathic isolated optic neuritis patients. We retrospectively analyzed 68 eyes of 59 patients diagnosed with optic neuritis and showed inflammatory findings indicative of optic neuritis on MRI. We investigated clinical features, such as the presence of accompanying pain, visual acuity, and optic disc swelling. Optic disc swelling was classified as normal, mild, or severe. The MRI results were divided into intraorbital, intracanalicular, and whole optic nerve according to the lesion, and these were compared and analyzed with clinical features. The study included 29 men and 30 women, with a mean age of 42.6 ± 16.6 years. Among 59 patients, 48 (81.4%) complained of pain. Optic disc swelling was not observed in 48.5% of patients (33 eyes). Inflammatory changes were the most common in the intraorbital region (33 eyes), intracanalicular region (20 eyes), and the entire optic nerve (15 eyes). There was no statistical difference in the pain pattern according to the location of the lesion (*p* = .677), but when inflammation was present in the entire optic nerve, optic disc swelling was severe (*p* = .023). The initial and final visual acuity did not significantly correlate with the MRI pattern, presence of pain, or optic disc swelling (*p* = .156, *p* = .714, and *p* = .436). The MRI contrast enhancement pattern was associated with optic disc swelling but was not associated with pain or initial visual acuity. It should be noted that it is insufficient to judge the clinical features of optic neuritis based on MRI findings.

## Introduction

Optic neuritis is a demyelinating optic nerve disorder that gives rise to visual disturbance and pain^1^. An optic neuritis treatment study (ONTT) is a monumental paper that provides valuable information about optic neuritis^2^. However, the ONTT study did not include patterns of magnetic resonance imaging (MRI) findings, and this study also has subtle differences from other ethnic group studies^3–5^.

The optic nerve cannot be visualized directly; thus, imaging studies, including MRI and computerized tomography, are required for diagnosis. MRI plays a crucial role in diagnosing optic neuritis and ruling out other optic nerve disorders. Sixty to 80% of patients with optic neuritis show signal alterations of the optic nerve on MRI^6–8^. Anatomical investigations are potent tools in predicting outcomes and clinical characteristics. Additionally, various conditions that develop optic neuritis show different patterns on MRI^9,10^.Therefore, this study aimed to investigate the characteristics of clinical features based on MRI in patients with idiopathic isolated optic neuritis.

## Methods

We retrospectively analyzed patients diagnosed with optic neuritis and showed inflammatory findings indicative of optic neuritis on MRI. The MRI findings were obtained within 2 weeks after onset. We excluded optic neuropathies related to systemic disorders including multiple sclerosis, neuromyelitis optica, and other central nervous system inflammatory disorders such as acute disseminated encephalomyelitis to minimize the impact on other diseases. Patients with abnormal chest X-ray or optic perineuritis were also excluded. Finally, 68 eyes of 59 patients who were able to follow up for more than 6 months were included.

All experimental protocol was approved by the Institutional Review Board of Kim’s Eye Hospital and conducted in accordance with the tenets of the Declaration of Helsinki. All methods were carried out in accordance with relevant guidelines and regulations. The IRB of Kim’s Eye Hospital allowed to waive the requirement to obtain the informed consent form because of minimal risk of this study.

**Figure 1:**
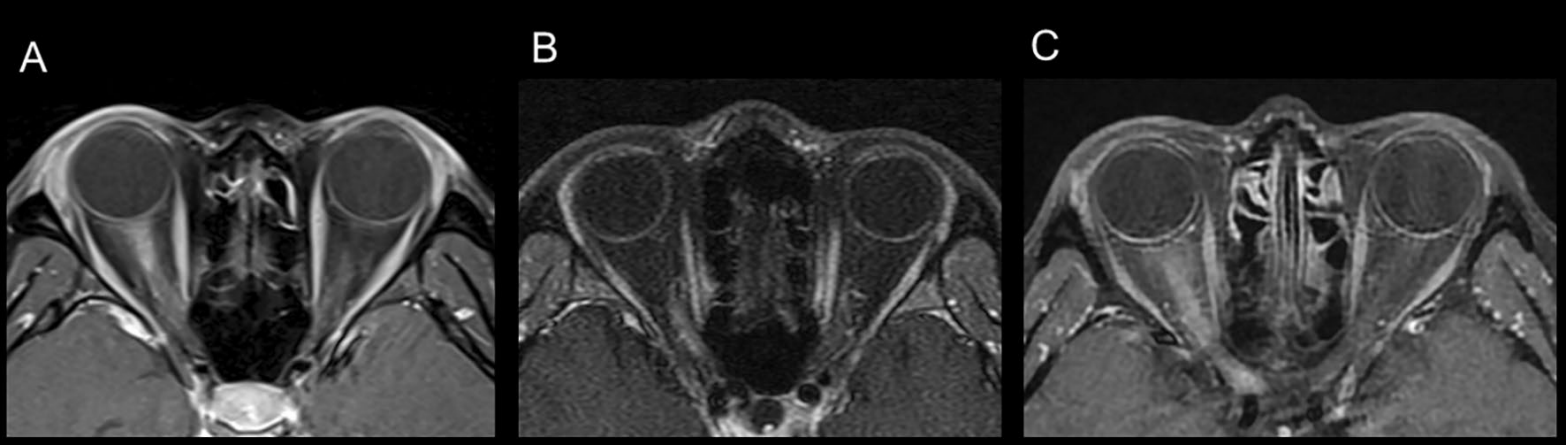
Location of abnormal findings on MRI. (**A**) intraorbital lesion, (**B**) intracanalicular area, (**C**) whole optic nerve involvement (optic nerve to canal).

Lesions detected on the MRI results were divided into intraorbital, intracanalicular, and whole optic nerve according to the contrast enhancement pattern (Fig. 1). When the lesion extended from the intraorbital to the intracanalicular area, it was designated as an intracanalicular group. Abnormal MRI findings were defined as high signals on T2 imaging, FLAIR imaging, or T1-Gd enhancement imaging. We investigated clinical features, such as pain, visual acuity (logMAR scale), and optic disc swelling. Optic disc swelling was classified as no optic disc swelling, mild, or severe according to the Frisen stage (stage 0, normal, stage 1–2; mild, and stage 3–5; severe)^11^. Initial visual acuity was checked within two weeks and before steroid therapy. The MRI findings were compared and analyzed for clinical features.

The data were analyzed using SPSS 26.0 for Windows (SPSS Inc., Chicago, IL, USA). Pearson’s chi-square test and one-way analysis of variance (ANOVA) were used to evaluate the relationship between MRI findings and clinical features.

## Results

The study included 29 males and 30 females, and the mean age was 42.6 ± 16.6 years (Table 1). Among 59 patients, 48 (81.4%) complained of pain. Optic disc swelling was not observed in 33 eyes (48.5%), mild in 17 eyes, and severe in 18 eyes.

**Table 1:** Demographics. Gender did not differ between the three groups (*p* = .596, Chi-square test).

	Male	Female	Age (years)
Intraorbital	14	19	45.0 ± 3.1
Intracanalicular	11	9	40.0 ± 4.0
Whole optic nerve	6	9	42.3 ± 2.9
Total	31	37	42.6 ± 16.6

Age was also similar across the three groups (*p* = .474, one-way ANOVA).

Nine patients showed bilateral involvement. The most common location of abnormal findings on MRI was in the intraorbital region (33 eyes, 47.8%), followed by the intracanalicular area (20 eyes), and whole optic nerve involvement (15 eyes). The age distribution did not differ between the three groups (*p* = 0.474). There was no statistical difference in the pain pattern according to the location of the lesion (*p* = 0.673) (Table 2), but when enhancement was applied to the whole optic nerve, optic disc swelling was severe (*p* = 0.023) (Table 3).

**Table 2:** The pattern of ocular pain depending on the location of lesions on MRI.

	No pain	Ocular pain	Total
Intraorbital	4	24 (85.7%)	28
Intracanalicular	5	13 (72.2%)	18
Whole optic nerve	2	11 (84.6%)	13
Total	11	48	59

Ocular pain was noted in the 48 patients (81.4%). However, there was no statistical difference among the three groups (*p* = .673, Chi-square test).

**Table 3:** Relationship between lesion location on MRI and optic disc swelling.

	Normal	Mild	Severe	Total
Intraorbital	16	10	7	33
Intracanalicular	12	6	2	20
Whole optic nerve	5	1	9	15
Total	33	17	18	68

Patients with lesions in the whole optic nerve tended to have severe optic disc swelling (*p* = .023, Chi-square test).

**Figure 2:**
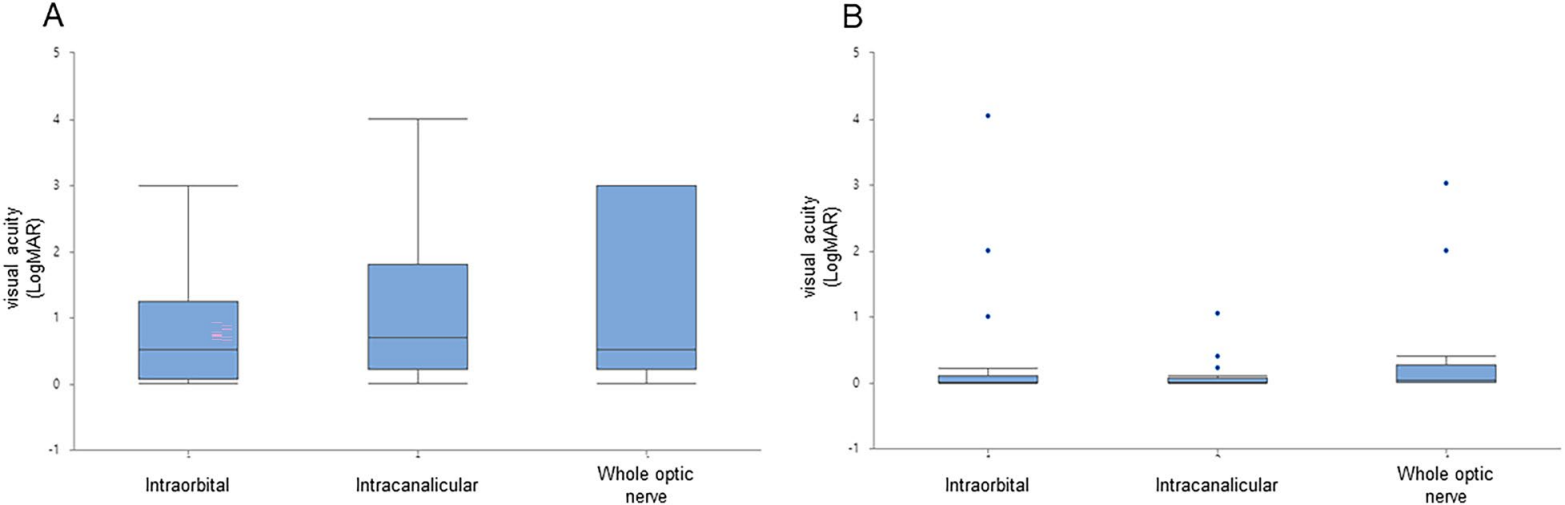
Visual acuity depending on the location of lesions on MRI. (**A**) visual acuity at initial visit (intraorbital; 0.83 ± 0.16, intracanalicular; 1.18 ± 0.27 and whole optic nerve; 1.15 ± 0.32) (*p* = .447). (**B**) Visual acuity at final follow-up (intraorbital; 0.28 ± 0.82, intracanalicular; 0.12 ± 0.25 and whole optic nerve; 0.45 ± 0.94) (*p* = .457).

**Figure 3:**
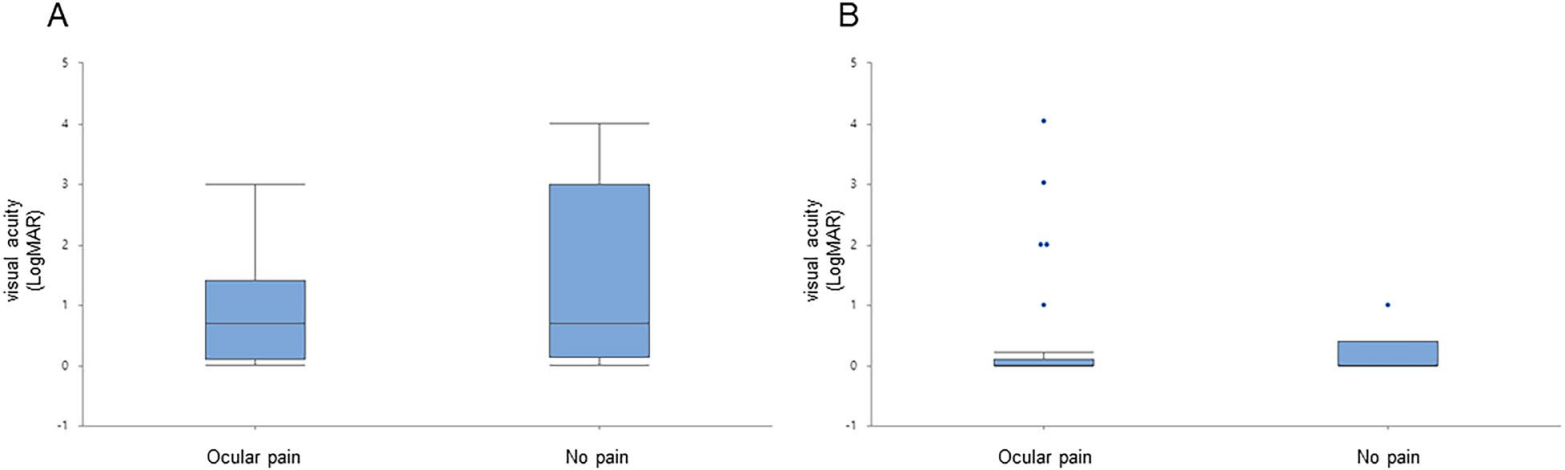
Visual acuity depending on the presence of ocular pain. (**A**) Visual acuity at initial visit (ocular pain group; 0.93 ± 0.13 and normal group; 1.30 ± 0.40) (*p* = .282). (**B**) Visual acuity at final follow-up (ocular pain group; 0.26 ± 0.12 and normal group; 0.31 ± 0.11) (*p* = .839).

The initial visual acuity and final visual acuity did not significantly correlate with the MRI pattern (*p* = 0.447 and *p* = 0.457) (Fig. 2). Final visual acuity in patients with intracanalicular lesions (0.12 ± 0.06) was better than in patients with other optic disorders; however, the difference was not statistically significant (*p* = 0.457). The initial visual acuity and the visual outcome did not differ significantly in the presence of pain (Fig. 3).

Fifteen of the 59 patients received corticosteroid therapy; however, this treatment did not affect the visual outcome in each group *(p* = 0.658, one-way ANOVA test).

## Discussion

In the present study, we investigated the association between MRI findings and clinical features of optic neuritis. Although optic disc swelling tends to occur severely when contrast enhancement is seen in the whole optic nerve on MRI, a relationship between MRI findings and clinical features is generally not found.

The Optic Neuritis Treatment Trial (ONTT) reported that approximately 90% of patients had ocular pain related to optic neuritis. The present study showed a slightly lower pain rate (81.4%), which could be related to ethnic characteristics because Japanese and Korean has previously demonstrated a lower rate of ocular pain than the ONTT study^3,12^. In terms of optic disc swelling, disc edema in the present study (approximately half) was noted to be higher than that in the ONTT study (one-third). Optic disc swelling is slightly more frequent in Asians, and the present result was similar to that of a Japanese study (50%),^12^ and somewhat less than that of a Korean study (59.3%)^13^.

In the present study, the intraorbital area was the most frequent lesion location in optic neuritis, which corresponds well with a previous study^8,14^. We excluded various CNS-related optic neuritises in this study, such as neuromyelitis optica (NMO), multiple sclerosis, and acute demyelinating disseminated myelitis, because these diseases have different outcomes. In our cohort, all patients who have enhancement limited to the chiasmal area were diagnosed with NMO and multiple sclerosis. As a result, focal lesions in the chiasm were not observed in this study.

The correlation between lesions on MRI and visual acuity was not defined. In the present study, visual acuity was not significantly correlated with the MRI findings. Berg et al.^6^ reported that lesion length on Gd-enhancement and T2WI has a mild correlation with the improvement of visual acuity; however, visual outcome does not differ depending on the lesion. In addition, another study on optic neuritis with multiple sclerosis showed that visual outcome was not related to MRI findings^15^. However, a recent study^16^ suggested that the length of the optic nerve lesion could be a predictor of chronic visual loss. The inconsistent results are probably a result of the many different causes of optic neuritis.

The MRI pattern was associated with optic disc swelling but not with pain or initial visual acuity. Ocular pain in optic neuritis is thought to originate from the inflamed optic nerve sheath at the orbital apex^17^. Therefore, the anterior part of the optic nerve could have a lesser effect on the development of pain. According to this hypothesis, the intracranial area is less likely to cause ocular pain; unfortunately, there were no cases involving only the intracranial area in this study. The present study showed that intracanalicular lesions had the lowest incidence of ocular pain. Hilary et al. ^18^ reported that optic neuritis involving the orbital segment frequently causes pain and pain with eye movement. Therefore, lesions around the orbital apex could be related to ocular pain in optic neuritis. However, we ran post-hoc analysis, and the power is not sufficient (0.37). Thus, a further large study is needed to clear the correlation.

This study has several limitations. First, we did not analyze optic neuritis caused by secondary lesions such as NMO or multiple sclerosis to improve the homogeneity of characteristics. In case of long-term follow-up, the possibility that related diseases may change cannot be excluded. Secondly, this was a retrospective review of charts, and some cases were excluded because of a lack of information. In addition, we did not test MOG antibody test on whole patients. This could be one of the confounding factors to be analyzed. Finally, other optic neuropathies could be included in the study, for example, early stage of multiple sclerosis or neuromyelitis optica.

In conclusion, it is insufficient to judge the clinical features of optic neuritis based on MRI findings. Therefore, when managing patients, various factors should be considered.

## Data Availability

Data used to support the findings of this study are available from the corresponding author upon request.
